# One-year outcomes of catheter ablation for atrial fibrillation in young patients

**DOI:** 10.1186/s12872-022-03017-6

**Published:** 2023-02-11

**Authors:** Andrew S. Tseng, Harsh P. Patel, Ashish Kumar, Chinmay Jani, Kirtenkumar Patel, Rahul Jaswaney, Samarthkumar Thakkar, Narayan G. Kowlgi, Sourbha S. Dani, Shilpkumar Arora, Siva K. Mulpuru, Malini Madhavan, Ammar M. Killu, Yong-mei Cha, Christopher V. DeSimone, Abhishek Deshmukh

**Affiliations:** 1 Department of Cardiovascular Diseases, Mayo Clinic, Rochester, MN USA; 2 Department of Cardiovascular Disease, Southern Illinois University School of Medicine, Springfield, IL USA; 3 Department of Internal Medicine, Cleveland Clinic Akron General, Akron, OH USA; 4 Department of Internal Medicine, Mount Auburn Hospital, Harvard Medical School, Cambridge, MA USA; 5 Department of Cardiology, North Shore University Hospital, Manhasset, NY USA; 6 Department of Medicine, Case Western Reserve University, Cleveland, OH USA; 7 Department of Internal Medicine, Rochester General Hospital, Rochester, NY USA; 8 Division of Cardiology, Lahey Hospital and Medical Center, Beth Israel Lahey Health, Burlington, MA USA; 9 Department of Cardiology, University Hospitals Cleveland Medical Center, Case Western Reserve University, Cleveland, OH USA; 10 Mayo Clinic College of Medicine, 200 1St St SW, Rochester, MN 55905 USA

**Keywords:** Atrial fibrillation, Catheter ablation, Ischemic stroke, Young, Readmission

## Abstract

**Background:**

Atrial fibrillation (AF) is relatively less frequent in younger patients (age < 50). Recently, studies have suggested that early restoration of sinus rhythm may lead to improved outcomes compared with rate control, however the efficacy of catheter ablation for AF in young is scarce.

**Methods:**

We included all hospitalized patients between 18 and 50 years with a diagnosis of AF from the Nationwide Readmission Database 2016–2017 from the Healthcare Cost and Utilization Project. Demographic and comorbidity data were collected and analyzed. Outcomes assessed included one-year AF readmission rates, all-cause readmission, ischemic stroke, and all-cause mortality. Subgroup analyses were performed for all demographic and comorbidity variables.

**Results:**

Overall, 52,598 patients (medium age 44, interquartile range 38–48, female 25.7%) were included in the study, including 2,146 (4.0%) who underwent catheter ablation for AF. Patients who underwent catheter ablation had a significantly lower rate of readmission for AF or any cause at one year (adjusted hazard ratios (HR) of 0.52 [95% confidence interval (CI): 0.43–0.63] and HR of 0.81 [95% CI: 0.72–0.89], respectively). There was no difference in 1-year readmission for stroke or all-cause mortality between the two groups. Subgroup analyses showed a consistent reduction in the risk of AF readmission among major demographic and comorbidity subgroups.

**Conclusion:**

Catheter ablation in young patients with AF was associated with a reduction in 1-year AF related and all-cause readmissions. These data merit further prospective investigation for validation, through dedicated registries and multicenter collaborations to include young AF from diverse population.

**Supplementary Information:**

The online version contains supplementary material available at 10.1186/s12872-022-03017-6.

## Background

Atrial fibrillation (AF) is the most common arrhythmia affecting millions of patients worldwide. It affects elderly patients with a greater than 10% prevalence after age 80 and those with pre-existing structural heart disease [1]. In this population, AF is associated with significant morbidity from ischemic stroke and congestive heart failure (CHF) [2]. However, AF can occur in relatively younger patients in the presence or absence of congenital or other structural heart diseases but still confers additional risk to patients, particularly the risk of poor quality of life, increased rate of hospitalization and healthcare utilization, ischemic stroke, and CHF [1, 3].

As AF is relatively infrequent in this population, our understanding of management and outcomes in the young is primarily extrapolated from studies with older patients. With increasing detection due to widespread technological and technical advances, such as smart devices with built-in electrocardiograms (ECGs), there will likely be an increasing number of young patients with AF [4]. It is particularly true as younger patients worldwide have an increasing burden of chronic illnesses, such as diabetes and hypertension, that may influence both the development of AF and the resultant cardiovascular sequelae [5, 6]. Furthermore, the EAST AF trial has shown that early rhythm control was associated with a decreased risk of cardiovascular mortality, stroke, and hospitalization with worsening CHF or acute coronary syndrome [7]. Thus, there is likely an increased enthusiasm to consider AF ablation, particularly in younger patients.

Catheter ablation for AF in young patients has previously been safe and efficacious, with high procedural success rates and low complication rates [8–11]. We present from the Nationwide Readmission Database (NRD) the one-year outcomes in young patients with AF undergoing ablation.

## Methods

### Study design

The study utilized the patient cohort from the publicly-available NRD, a subset of the Healthcare Cost and Utilization Project (HCUP) from the Agency for Healthcare Research and Quality (AHRQ), from 2016 to 2017 (https://www.hcup-us.ahrq.gov/nrdoverview.jsp). The NRD from 2016 to 2017 contains data from approximately 17 million discharges from 26 states, accounting for nearly 58.2% of all hospitalizations in the United States. Given the study's nature utilizing publicly-available de-identified data only, the study was exempt from institutional review board review.

### Baseline characteristics

**Fig. 1:**
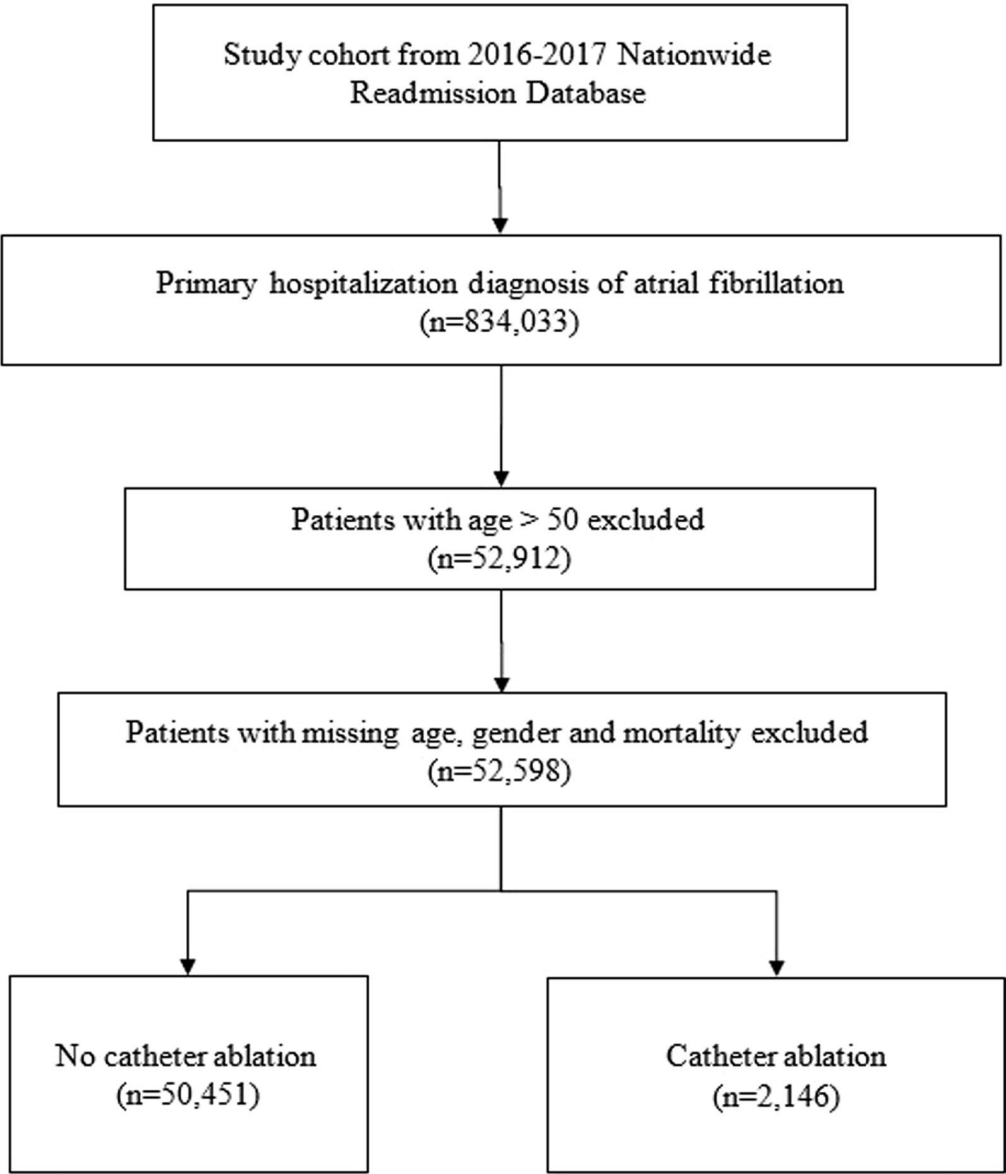
Flow diagram of cohort derivation. Flow diagram showing cohort derivation based on inclusion and exclusion criteria

We included all patients in this dataset between the ages of 18 and 50 with AF diagnosis. Patients with AF were identified using ICD-10 CM, International Classification of Diseases, 10^th^ Revision, Clinical Modification codes (ICD-10 CM: I48.0, I48.1, I48.2, I48.91) as a primary or secondary diagnosis (Additional file 1: Table S1) [12]. These patients were further subdivided into patients who underwent catheter ablation for AF during the index hospitalization and those that did not. Catheter ablation was defined using ICD-10 CM codes, 02563ZZ, and 02583ZZ (Additional file 1: Table S1) [12, 13]. The flow diagram for the cohort derivation is shown in Fig. 1**.** The index hospitalization for the non-ablation group would be the first hospitalization in the year of NRD database. The index hospitalization for the ablation group would be the hospitalization during which the ablation was performed. The median follow up was determined by taking the median of the follow up period from index hospitalization (as previously described) till the end of the year of the respective NRD database or occurrence of morality.

Baseline demographic, socioeconomic, and hospitalization data were collected, including but not limited to age, sex, median household income, primary payer, admission day of the week, hospital size, etc. Comorbidities, including obstructive sleep apnea, obesity, hypertension, diabetes, coronary artery disease, chronic obstructive pulmonary disease, chronic kidney disease stage 3 or greater, prior coronary artery bypass grafting, hyperthyroidism, alcohol use disorder, prior stroke, prior transient ischemic attack, mitral valve stenosis, peripheral vascular disease, anemia, congestive heart failure were identified using ICD-10 CM codes. The ICD-10 codes used to define comorbidities in this analsyis are elaborated in Additional file 1: Table S1. For median household income, the representative quartile of median household income of residents within the residential zip code was used, derived from zip code demographic data obtained from Claritas (Claritas LLC, Cincinnati, Ohio). The quartiles are identified from values of 1 to 4, indicating lowest to the highest quartile. Note that these estimates are updated annually, and value ranges can vary by year (https://www.hcupus.ahrq.gov/db/vars/zipinc_qrtl/nrdnote.jsp). The bed size cutoff points divided into small, medium, and large have been done so that approximately one-third of the hospitals in a given region, location, and teaching status combination would fall within each bed size category (https://www.hcupus.ahrq.gov/db/vars/hosp_bedsize/nrdnote.jsp). A hospital is considered a teaching hospital if it has an American Medical Association-approved residency program, is a member of the Council of Teaching Hospitals, or has a ratio of full-time equivalent interns and residents to beds of 0.25 or higher (https://www.hcupus.ahrq.gov/db/vars/hosp_ur_teach/nrdnote.jsp). The CHA_2_DS_2_VASc score was calculated using t comorbidity data provided in Additional file 1: Table S1.

### Outcomes

The outcomes of interest were 1-year all-cause hospital readmission, 1-year AF hospital readmission, 1-year ischemic stroke hospital readmission, and 1-year all-cause mortality. All-cause hospital readmission was defined as any readmission both with regards to numbers and reason for a patient, during the follow-up duration. Cause-specific hospital readmission was defined as any readmissions in which either AF or ischemic stroke was the primary diagnosis. The ICD-10 CM codes for ischemic stroke are included separately (Additional file 1: Table S1). The choice of endpoints was to mimic routinely reported endpoints of randomized controlled trails and other studies.

### Data analysis and statistics

Descriptive statistics were used to analyze the demographic and comorbidity data. We used the Chi-squared test to compare categorical variables and the Mann–Whitney U test to compare continuous variables. Time-to-event analysis was utilized using the Kaplan–Meier method and the Cox proportional hazard regression analyses. Two Cox proportional hazard models were utilized: one fully-adjusted for all significant demographic and comorbidity variables and one adjusted for variables part of the CHA_2_DS_2_VASC score. The log-rank test was used to generate p- values for Kaplan–Meier curves. Subgroup analysis was performed for each demographic and comorbidity variables for ablation versus no ablation assuming the comorbidity of interest was present. A two-tailed p-value of < 0.05 was considered statistically significant. The data analysis was performed using SAS 9.4 (SAS Institute Inc., Cary, North Carolina), and SPSS 26 (IBM Corporation, Chicago, Illinois) was used for statistical analysis.

## Results

### Baseline cohort characteristics

A total of 52,598 patient (median age 44[IQR: 38–48], and female 25.7%) were included in the study, with a median follow-up duration of around 183 days. A total of 2,146 (4.0%) patients underwent catheter ablation. Compared with patients who did not undergo catheter ablation, patients who underwent ablation were notably more likely to be older (45 (40–48) vs. 44 (38–48), *P*-value < 0.001) and had a higher proportion of male (78.3% vs. 74.2%, *P*-value < 0.001). These patients had a higher median household income, private insurance, and a more significant proportion of elective hospitalizations to large teaching hospitals. The demographic data are summarized in Table 1.

**Table 1:** Baseline Characteristics of patients with atrial fibrillation

Atrial Fibrillation patients	No Ablation	Ablation	Overall	*P*-value
n	50,451	2146	52,598	
Age (Years) (Median(IQR))	44 (38–48	45 (40–48)	44 (38–48)	< 0.001
*Age Group*				< 0.001
18–29	8.4%	5.5%	8.2%	
30–39	22.3%	16.1%	22.0%	
40–50	69.4%	78.4%	69.7%	
*Gender*				< 0.001
Male	74.2%	78.3%	74.3%	
Female	25.8%	21.7%	25.7%	
*Comorbidities*				
OSA	18.9%	26.3%	19.2%	< 0.001
Obesity	36.5%	32.7%	36.3%	< 0.001
Hypertension	53.6%	51.8%	53.5%	0.100
Diabetes	17.6%	15.5%	17.5%	0.010
Coronary Artery Disease	13.5%	13.0%	13.5%	0.530
COPD	5.3%	5.3%	5.3%	0.970
CKD stage 3 or more	7.5%	6.7%	7.4%	0.170
Prior CABG	1.0%	1.2%	1.0%	0.490
Hyperthyroidism	3.3%	1.3%	3.2%	< 0.001
Alcohol Disorder	12.9%	3.9%	12.5%	< 0.001
Mitral Valve Stenosis	0.3%	0.2%	0.3%	0.230
Prior Stroke/TIA	3.9%	4.9%	3.9%	0.010
Peripheral vascular disease	0.9%	0.9%	0.9%	0.920
Anemia	8.8%	7.4%	8.8%	0.030
Heart Failure	0.2%	0.1%	0.2%	0.480
*CHA*_*2*_*DS*_*2*_*VASc Score*				0.02
0	31.0%	34.6%	31.1%	
1	41.9%	40.4%	41.9%	
2	19.4%	17.3%	19.3%	
3	5.9%	6.1%	5.9%	
4	1.4%	1.2%	1.4%	
5	0.4%	0.3%	0.4%	
> = 6	0.01%	0.00%	0.01%	
*Median household income category for patient’s zip code*				< 0.001
0-25th percentile	32.1%	23.5%	31.7%	
26-50th percentile	27.2%	23.5%	27.0%	
51-75th percentile	23.9%	24.9%	23.9%	
76-100th percentile	16.9%	28.0%	17.3%	
*Primary Payer*				< 0.001
Federal insurance	30.4%	24.7%	30.1%	
Private insurance	69.6%	75.2%	69.9%	
*Hospital characteristics*				
*Hospital bed size*				< 0.001
Small/Medium	46.8%	27.0%	46.0%	
Large	53.1%	73.0%	54.0%	
*Hospital teaching status*				< 0.001
Non-Teaching	37.2%	14.8%	36.3%	
Teaching	62.8%	85.2%	63.7%	

*IQR* interquartile range, *OSA* obstructive sleep apnea, *COPD* chronic obstructive pulmonary disease, *CKD* chronic kidney disease, *CABG* coronary artery bypass graft, *TIA* transient ischemic attack

### Outcomes at one year

**Fig. 2:**
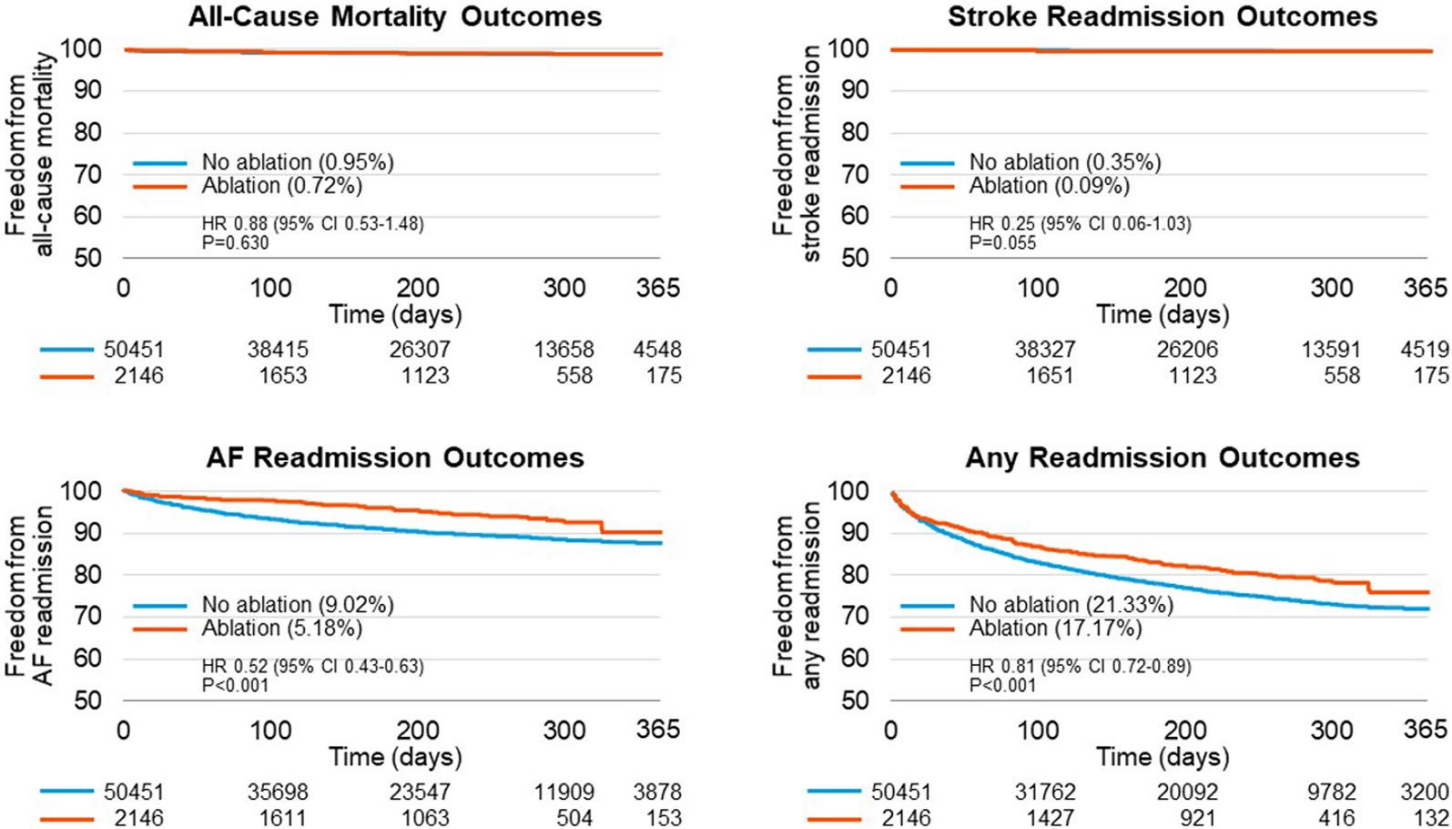
Kaplan–Meier curves of outcomes of interest by ablation status. The Kaplain-Meier curves for freedom from all-cause mortality, stroke readmission, AF readmission, and any readmission is shown, where the blue curve represents patients without ablation and red curve represents patients with ablation. The number at risk at each major time point is provided. The adjusted hazard ratios are provided

Overall, patients who underwent ablation had a lower risk of readmission for AF or any cause at one year, with an adjusted HR of 0.52 (95% CI: 0.43–0.63) and 0.81 (95% CI: 0.72–0.89), respectively. One-year readmission rates for AF were 5.2% and 9.0% for patients with ablation and without ablation, respectively. For any cause, one-year readmission rates were 21.3% and 17.2% for patients without ablation and with ablation, respectively. There was no difference in 1-year readmission for stroke or all-cause mortality between the two groups. The data is summarized in Table 2, and event-free survival is shown in Fig. 2.

**Table 2:** Adjusted hazard ratios for primary and secondary outcomes at 1-year

	No ablation (n = 50,541)	Ablation (n = 2146)	p-value
*Fully-Adjusted model* †			
AF readmission	9.02%	5.18%	
AF readmission (HR, 95% CI)	0.52 (0.43–0.63)		< 0.001
Any readmission	21.33%	17.17%	
Any readmission (HR, 95% CI)	0.81 (0.72–0.89)		< 0.001
Stroke readmission	0.35%	0.09%	
Stroke readmission (HR, 95% CI)	0.25 (0.06–1.03)		0.055
All-cause mortality	0.95%	0.72%	
All-cause mortality (HR, 95% CI)	0.88 (0.53–1.48)		0.630
*CHA*_*2*_*DS*_*2*_*VASc Score-Adjusted Model*‡			
AF readmission (HR, 95% CI)	0.56 (0.46–0.67)		< 0.001
Any readmission (HR, 95% CI)	0.79 (0.71–0.88)		< 0.001
Stroke readmission (HR, 95% CI)	0.27 (0.07–1.11)		0.07
All-cause mortality (HR, 95% CI)	0.78 (0.47–1.30)		0.34

^†^ Individual cox proportional hazard regression models were run for each outcome, all models were adjusted for age, gender, OSA, Obesity, hypertension, diabetes, coronary artery disease, COPD, CKD stage 3 or more, prior CABG, hyperthyroidism, alcohol disorder, mitral valve stenosis, prior stroke/TIA, peripheral vascular disease, anemia, heart failure, median household income, primary payer, hospital bed size, and hospital teaching status

^‡^ Individual cox proportional hazard regression models were run for each outcome, all models were adjusted for CHA_2_DS_2_VASC score

Subgroup analyses showed a consistent reduction in AF readmission risk among major demographic and comorbidity subgroups, except age between 18 and 29, anemia, and prior CABG. In particular, AF ablation in both male and female patients and AF ablation performed at teaching institutions showed a reduced risk of AF readmission. Patients with lower CHA_2_DS_2_VASc scores (≤ 4) had a reduced risk of AF readmissions. Two hundred patients (0.4%) had scores greater than 4. The data is summarized in Table 3.

**Table 3:** Subgroup Analysis of Atrial Fibrillation readmission

Atrial Fibrillation readmission		Ablation			
Subgroups	*n*	HR	LL	UL	*p*-value
*Age Group*					
18–29	4339 (8.25%)	1.37	0.64	2.91	0.41
30–39	11578 (22.01%)	0.35	0.17	0.69	0.003
40–50	36680 (69.74%)	0.54	0.44	0.67	< 0.001
*Gender*					
Male	39100 (74.34%)	0.53	0.43	0.66	< 0.001
Female	13498 (25.66%)	0.55	0.38	0.8	0.001
*Comorbidities*					
OSA	10079 (19.16%)	0.48	0.34	0.69	< 0.001
Obesity	19095 (36.3%)	0.50	0.37	0.68	< 0.001
Hypertension	28137 (53.49%)	0.49	0.38	0.63	< 0.001
Diabetes	9213 (17.52%)	0.39	0.24	0.62	< 0.001
Coronary Artery Disease	7102 (13.5%)	0.33	0.19	0.58	< 0.001
COPD	2797 (5.32%)	0.23	0.09	0.51	0.0004
CKD stage 3 or more	3912 (7.44%)	0.36	0.18	0.7	0.003
Prior CABG	544 (1.03%)	0.51	0.09	2.74	0.44
Hyperthyroidism	1677 (3.19%)	0	0	0	0.98
Alcohol Disorder	6589 (12.53%)	0.13	0.02	0.68	0.02
Mitral Valve Stenosis	160 (0.3%)	N/A	N/A	N/A	N/A
Prior Stroke/TIA	2067 (3.93%)	0.13	0.03	0.54	0.005
Peripheral vascular disease	488 (0.93%)	N/A	N/A	N/A	N/A
Anemia	4613 (8.77%)	0.55	0.28	1.06	0.07
Heart Failure	101 (0.19%)	N/A	N/A	N/A	N/A
*CHA*_*2*_*DS*_*2*_*VASc Score*					
0	16382 (31.1%)	0.64	0.44	0.91	0.01
1	22028 (41.9%)	0.67	0.51	0.88	0.004
2	10141 (19.3%)	0.43	0.27	0.67	0.0002
3	3128 (5.9%)	0.31	0.13	0.73	0.007
4	719 (1.4%)	0.38	0.09	1.56	0.18
5	197 (0.4%)	0.00	0	0	0.99
6	3 (0.01%)	n/a	n/a	n/a	n/a
*Primary Payer*					
Federal insurance	15854 (30.14%)	0.33	0.22	0.48	< 0.001
Private insurance	36739 (69.86%)	0.68	0.55	0.85	0.001
*Hospital characteristics*					
Hospital bed size					
Small/Medium	24214 (46.04%)	0.44	0.29	0.68	0.0002
Large	28383 (53.96%)	0.57	0.46	0.71	< 0.001
*Hospital teaching status*					
Non-Teaching	19109 (36.33%)	0.67	0.43	1.03	0.07
Teaching	33489 (63.67%)	0.52	0.42	0.64	< 0.001

*IQR* interquartile range, *OSA* obstructive sleep apnea, *COPD* chronic obstructive pulmonary disease, *CKD* chronic kidney disease, *CABG* coronary artery bypass graft, *TIA* transient ischemic attack

## Discussion

In the present study, we evaluated the one-year outcomes of AF ablation in a relatively young patient cohort (less than age 50) compared with those who did not undergo ablation in a large nationwide database analysis from 52,598 patients. The principal findings in our study were: (1) young patients who underwent catheter ablation for AF had lower readmission rates for AF, stroke, and any cause, (2) there was no difference in mortality at one year, and (3) there were significant demographic, socioeconomic, and comorbidity differences in patients who underwent catheter ablation compared with those who did not.

Previous studies have suggested that catheter ablation may be a favorable therapeutic option for young patients with AF, citing high procedural success rates and low complication rates. Our findings are consistent with these observations as we found a reduction in readmission rates for AF and any cause, but no difference in all-cause mortality. It may be due to various factors, including freedom from AF, fewer symptomatic recurrent AF episodes, closer outpatient follow-up after ablation, and concurrent use of antiarrhythmics in the post-ablation period [8–11] (summary of studies shown in Table 4). Our study reported lower recurrence rate overall compared with studies presented in Table 4, which could be explained because of better ablation techniques, early diagnosis and experience with the use of catheter ablation for AF. In this study, subgroup analysis showed a consistent reduction in the risk of one-year AF readmissions for most patient subgroups who underwent catheter ablation. These results are similar to other studies, including the CABANA trial [14].

**Table 4:** Summary of select studies of AF ablation in young patients

Study, year	Sample size	Mean age (SD)	Follow-up duration	Outcome
Dewire et al., 2013 [8]	40	34.1 (5.6)	3.8 (2.9) years (mean, SD)	62.5% free of AF without antiarrhythmic drugs 100% with > 95% reduction of AF burden on or off AADs
Saguner et al., 2018 [9]	85	31 (4)	4.6 (4.0) years (median, IQR)	84% in stable SR Single procedure 44% 5-year arrhythmia–free survival
Leong-Sit et al., 2010 [10]	232	< 45	32 months	87% with ≤ 6 AF episodes over the follow-up year that terminated either spontaneously and/or with a single cardioversion and/or a > 95% reduction in AF burden
Chun et al., 2013 [11]	593	41 (38–44) (median, IQR)	12 months	36.2% all-cause re-admission rate 17.6% repeat ablation rate 1.4% death, MI or stroke

Our study also shows contemporary patterns in patient selection for AF ablation among younger patients, namely those with fewer comorbidities for ablation. After ablation, predictors of AF recurrence appear to be similar to that of older patients with AF, including obesity and structural heart disease [15, 16]. There is substantial evidence that clinical stratification plays a vital role in determining referral for ablation and procedural outcomes of ablation for any patient with AF. In our population, we note those with obesity were less likely to undergo ablation procedure during the study period, likely due to the perceived risk of complications or increased recurrence rates after catheter ablation or initial emphasis on risk factor modification.

Social determinants, overall physical health, and comorbidity burden do likely lead to selection bias while offering AF ablation [15]. We found disparities in the patient selection based on socioeconomic status, with a more significant proportion of patients in the upper quartile of income being referred for ablation. Lower rates of catheter ablation in non-whites and less affluent patients have previously been shown. It may reflect healthcare availability and access to healthcare resources, as shown previously [17]. There may also be a relationship between socioeconomic status and predictors of AF recurrence, such as obesity, that may have impacted patient selection [18].

Nonetheless, even patients with comorbidities such as obesity and alcohol abuse had a lower risk of AF readmission with AF ablation compared with no ablation on subgroup analysis. Patients were also more likely to have undergone ablation in larger teaching hospitals, reflecting facilities' availability, and experienced proceduralists with higher volumes of ablations [19]. Thus, patient selection and procedures in presumably higher volume centers (i.e., larger teaching hospitals) may contribute to the positive post-ablation outcomes seen in this study, which as noted previously was better as compared with the previously published literature.

### Limitations

There were limitations inherent in the study design and available data. First, we were only able to evaluate one-year outcomes for hospitalized patients. Patients seen in the primary care office, emergency department alone, or under observation, were not included in this database. As such, many healthier young patients with AF may be underrepresented in this study. These early benefits from catheter ablation may be attenuated in the long term.

Given that this was a retrospective study reliant on ICD codes, there is a possibility of misclassification, and coding accuracy is dependent on individual providers and institutions. Moreover, many patients had codes for unspecified AF, so analysis on AF type was unable to be performed. We could not obtain patient-level granular clinical information such as a history of symptom status, previous AF hospitalizations, or prior/concurrent therapies such as medications (particularly, anticoagulation and antiarrhythmic usage) or cardioversion. Procedural details like operator experience, ablation strategy, energy source, and procedural success rates were also unable to be obtained.

## Conclusion

Catheter ablation for AF in younger patients (age < 50 years) is associated with decreased risks of one-year AF readmission and all-cause readmission. However there was not difference in stroke readmissions or all-cause mortality between the two treatment groups. However, these findings must be validated in a randomized and long-term prospective analysis.

## Supplementary Information

**Additional file 1.** Supplemental table for International Classification of Diseases 10th Revision codes utilized in this study.

## Data Availability

All data generated or analyzed during this study are included in this published article [and its additional information files]. The data can accessed at https://www.distributor.hcup-us.ahrq.gov/Databases.aspx.

## Abbreviations

AHRQ: Agency for healthcare research and quality
AF: Atrial fibrillation
HCUP: Healthcare cost and utilization project
ICD-10-CM: International Classification of Diseases, 10th Revision, Clinical Modification
IQR: Interquartile range
NRD: Nationwide readmission database
