# Supplementary material for: One-year outcomes of catheter ablation for atrial fibrillation in young patients
Source: BMC Cardiovasc Disord. 2023 Feb 11;23:83. doi: 10.1186/s12872-022-03017-6 (PMC9921413; doi:10.1186/s12872-022-03017-6)
Supplement: Supplementary file 1 — Additional file 1. Supplemental table for International Classification of Diseases 10th Revision codes utilized in this study. [file 12872_2022_3017_MOESM1_ESM.docx]

|  | **ICD 10 codes** |
| --- | --- |
| **Atrial fibrillation** | I48.0, I48.1, I48.2, I48.91 |
| **Ablation (procedural code)** | 02563ZZ, 02583ZZ |
| **Comorbidities** | I20, I21, I22, I23, I24, I25 |
| OSA | G47.33 |
| Obesity | E66, Z68.3, Z68.4 |
| Hypertension | I10, I11, I12, I13, I14, I15, I16 |
| Diabetes | E08, E09, E10, E11, E13 |
| Coronary Artery Disease | I20, I21, I22, I23, I24, I25 |
| COPD | J41, J42, J43, J44 |
| CKD stage 3 or more | N18.3, N18.4, N18.5, N18.6, E08.2, E09.2, E10.2, E11.2, E13.2, I12, I13 |
| Prior CABG | I25.70, I25.71, I25.72, I25.73, I25.76, I25.79, I25.810, I25.812, T82.21, Z95.1 |
| Hyperthyroidism | E05 |
| Alcohol Disorder | F10, Z71.40, K29.2, G31.2, K85.2, K86.0, T51, I42.6, K70, G62.1 |
| Mitral Valve Stenosis | I34.2, I05.0, I05.2 |
| Prior Stroke/TIA | I69.3, Z86.73 |
| Peripheral vascular disease | E08.5, E09.5, E10.5, E11.5, E13.5, I73, T82.856, Z98.62, Z95.820 |
| Anemia | D5x, D60, D61, D62, D63, D64, D46.0, D46.1, D46.2, D46.4, O99.0 |
| Heart failure | I11.0, I13.0, I13.2, I50 |
| All the ICD 10 codes were verified by 2 independent authors. | |

**Supplementary Table 1:** ICD-10 codes used in the study.
